# Supplementary material for: Chromothripsis during telomere crisis is independent of NHEJ, and consistent with a replicative origin
Source: Genome Res. 2019 May;29(5):737–49. doi: 10.1101/gr.240705.118 (PMC6499312; doi:10.1101/gr.240705.118)
Supplement: Supplemental Material [file supp_gr.240705.118_Supplemental_file_1.zip › contigs/annotated_contigs/DB93/contig.2.DB93_length_322_mean_cov_5.56832298137.docx]

**DB93_length_322_mean_cov_5.56832298137**

```
ATGATGTGGAATATCACTCTCTTAAAAAATGCAAACAATTTATTTAATAGTCATAGTGCTAAAGATGGCATGCCCTCCAAATGAGCAAT
 >chr5:21514989-21515267 + E=3e-141
TGCACTCTTTGTTATGCATTTATATGAATATGTATATAGGAATAGTCATGATAAAACTACTAATTATTATAAAATTTTTGGAAAAAGCA

TAACTATTAAAAATGAAATTGGTAACTTAAGACTACTCAATACATCAACAAAAATGATTGAACTATGCAAAAACATGGATGAATTTCAA

AAATGA|AATTT|TTTTTTTTTTTTTGAGACGGAGTCTCGCTCTGTCGCCCAGGCTG       >chr11:57519965-57520014 - E=4e-18
```
